# Scopolamine-induced memory impairment in mice: Soursop leaf extract and fractions protect the hippocampus and prefrontal cortex

**DOI:** 10.22038/ijbms.2024.80289.17379

**Published:** 2025

**Authors:** Faith Afolabi, Babatunde Adebola Alabi, Joseph Ayo Badejo, Okim Okim Nsor, Sodiq Kolawole Lawal, Ezekiel Olugbenga Iwalewa

**Affiliations:** 1 Neuropharmacology Unit, Department of Pharmacology and Therapeutics, Faculty of Basic Medical Sciences, College of Medicine, University; 2 of Ibadan, Ibadan, Oyo State, Nigeria; 3 Department of Pharmacology & Therapeutics, Faculty of Basic Clinical Sciences, Bowen University, Iwo, Nigeria; 4 Department of Pharmacology & Therapeutics, Faculty of Medicine and Pharmacy, Kampala International University in Tanzania, Dar Es Salaam,; 5 United Republic of Tanzania; 6 School of Nursing, Faculty of Health Sciences, University of Botswana, Gaborone, Botswana

**Keywords:** Acetylcholinesterase, Glutamic acid, Inflammatory mediators, Memory impairment, Thin-layer chromatography

## Abstract

**Objective(s):**

*Annona muricata*” (soursop) is a medicinal plant with diverse ornamental, consumptive, and pharmacological importance. This study was designed for its anti-amnesic potential in mice.

**Materials and Methods:**

The crude extract was fractionated with n-hexane, ethyl acetate, and aqueous methanol. The crude and fractions were tested *in vitro *for the anti-oxidant radical scavenging activity (DPPH), total flavonoid and phenolic content, and their acetylcholinesterase inhibitory activity. Thin-layer chromatography was used to determine the phytochemicals contained in the fractions and their purity. Neurobehavioral models like the Open Field Test, Novel Object Recognition Test, Y-Maze, and Morris Water Maze were used to evaluate the action of AMME (*Annona muricata* methanol extract) and AMAMF (*Annona muricata *aqueous methanol fraction).

**Results:**

The AMME and AMAMF significantly reduced the serum levels of myeloperoxidase and arginine (*P*<0.05). They also modulate the hippocampal and prefrontal acetylcholinesterase and glutamic acid decarboxylase activities (*P*<0.05). The 25 mg/kg AMAMF significantly affected short-term memory (*P*<0.05). The AMAMF and AMME significantly enhanced the prefrontal and hippocampal tissue levels of glutathione and superoxide dismutase. During the *in vitro* AchE assay on all fractions, the AMME and AMAMF consistently showed the greatest percentage of inhibitory activity. They inhibited 50% of the AchE enzymes with the lowest concentration.

**Conclusion:**

The study showed the neuroprotective effects of AMME and AMAMF in memory impairment models. The extracts showed potent AChE inhibition and positive effects on memory and anti-oxidant enzyme levels.

## Introduction

The most prevalent type of dementia is Alzheimer’s disease (AD), a neurological condition that affects the brain and commonly leads to physical and cognitive impairment (1). AD is characterized by memory loss and is most common in elderly female individuals of 65 years and above (2). The World Alzheimer’s Organization Report from 2015 states that there are around 46.85 million people worldwide who suffer from AD and various types of dementia. This number is anticipated to double by 2030 and triple by 2050 (3).

Cognitive impairment does not only occur in the case of AD; studies have shown that amnesia, a deficit in memory and inability to learn new information as a result of brain damage and diseases, can also be a cause of loss in cognitive functions, which occurs majorly in case of severe amnesia. Amnesia is characterized by damage to specific areas of the brain, including the frontal lobe, thalamus, and hippocampus, which presents with personality changes such as apathy and poor judgment as seen in patients with frontal lobe lesion and atrophy in the case of Korsakoff’s syndrome (4). 

The neuropathological hallmark of AD-associated cognitive decline is the accumulation of pro-inflammatory mediators and reactive species. AD is closely linked to oxidative stress pathways like increased protein nitration and oxidation, glycoloxidation, and lipid peroxidation. Reduction in anti-oxidant levels also affects synaptic activity and neuronal transmission, leading to cognitive deficit (5). Non-steroid anti-inflammatory drug use (NSAIDs) could not demonstrate sufficient advantages in clinical practice, most likely because of the intricate connection between natural immunity and the pathophysiology of AD. Furthermore, anti-oxidant therapy may only provide theoretical protection against Aβ toxicity and oxidative stress. It is unreasonable to expect anti-oxidant strategies to be effective in stopping the advancement of AD on their own in clinical practice because oxidative stress is only one aspect of the intricate pathways leading to AD.

The cholinergic system regulates the neurotransmission pathways implicated in memory and learning, and alteration/obstruction of cholinergic activities is involved in the pathogenic process of AD (6). The process of neuronal degeneration of cholinergic neurons is followed by an obstruction in acetylcholine synthesis, which ultimately results in progressive impairment of memory and cognitive functions (7). Therefore, targeting cholinergic damage as a therapeutic intervention could be a means to improve memory and cognitive functions in AD individuals. Treatment options for cognitive impairment include N-methyl-D-aspartate receptor agonists and acetylcholinesterase inhibitors (8). Regrettably, side effects like vomiting, nausea, insomnia, and anorexia have been observed with the use of this class of drugs (1). Since plant phytochemicals have been shown to possess great potential against several diseases, including Alzheimer’s, without imposing significant adverse effects, we hypothesize using phytochemicals from the leaf of *Annuna muricata* (AM) against scopolamine-induced memory impairment in this study.

Different parts of AM have been employed in the conventional traditional treatment of malaria, cancer, diabetes, insomnia, cystitis, neuralgia, anxiety, and many others (9; 10). AM also possesses active compounds, including vitamins, alkaloids, acetogenins, carotenoids, and flavonoids (11). It is well known that flavonoids have a variety of beneficial benefits on the brain, such as the capacity to reduce neuroinflammation, shield neurons from damage brought on by neurotoxins, and enhance cognition, memory, and learning processes (12). Due to inadequate scientific information on the role of AM phytochemicals against memory impairment, we studied the ameliorative role played by AM leaf extract and fractions against scopolamine-induced memory impairment in mice.

## Materials and Methods

### Chemicals and drugs

The biochemical study utilized D-galactose (BDH Chemicals Ltd, Poole, England), ammonium sulfate, donepezil, Ellman reagent [5’,5’-dithiobis-(2-nitrobenzoate) DTNB], thiobarbituric acid (TBA), hyoscine butylbromide, trichloroacetic acid (TCA) from Burgoyne Burbidges & Co., Mumbai, India, hydrogen peroxide, acetylthiocholine, phosphate-buffered saline, distilled water, and all other analytical-grade chemicals.

### Plant extraction

Fresh leaves of *Annona muricata* were collected from the University of Ibadan’s botanical garden. They were identified and verified at the Forestry Research Institute of Nigeria’s Forest Herbarium, Department of Forest Herbarium, in Jericho, Ibadan, Oyo State, Nigeria, where the voucher specimen number FHI 112618 was assigned. After shade-drying, the leaves were ground into a powder using a high-capacity grinder and soaked in 70% aqueous methanol at a 1:10 (w/v) ratio. The resulting crude extract was then fractionated using n-hexane, ethyl acetate, and aqueous methanol. The crude and fractions were tested *in vitro* for the anti-oxidant radical scavenging activity (DPPH), total flavonoid and phenolic content, and their Acetylcholinesterase inhibitory activity. 

### Extraction fractioning protocol

500 g of dried, blended leaves were placed in a glass container (desiccator with lid) and submerged in 3,000 ml of pure methanol to obtain the extract. The mixture was stirred with a glass rod every two hours and left to sit for 72 hr. After this period, the solvent containing the extract was filtered using a muslin bag, and the filtrate was further clarified through Whatman filter paper (1mm). The extraction was repeated using an additional two liters of methanol to maximize the yield. The combined filtrate was concentrated at 40 °C with a rotary evaporator (Heidolph Laborota 400 efficient, made in Germany, model 517-01002-002) and air-dried on a wide petri dish to eliminate residual methanol. The percentage yield was then calculated.

Vacuum Liquid Chromatography (VLC) was used to purify the chloroform fraction further. Concentrated H_2_SO_4_ was used to thoroughly clean the previously cleaned sintered glass in Buchner to remove contaminants from the sieve. Next, three-quarters of the column was filled with silica gel 60 (0.040-0.063 mm, MERCK). After that, the column was mounted on a Buchner conical flask, and the vacuum pump was attached to it. After turning on the pump, the column was topically treated with n-hexane solvent. This was done to pack the column further. 12 g of the chloroform fraction was then adsorbed on 8 g of MERCK-grade silica gel 60 (0.040-0.063 mm). After stirring the gel-sample mixture, a homogeneous mixture was achieved. 

After the mixture was allowed to air dry and put to the top of the column, 100% n-hexane was added using the first solvent system while the pump was turned on. There was a 25 ml/min flow rate. Seven hundred fifty milliliters of the n-hexane solvent were used for this. The next solvent system, n-hexane: chloroform (1:1), was prepared by combining 50 milliliters of n-hexane with 50 ml of chloroform. The solvent system was then used to elute the column. This process continued until the fraction had wholly eluted from the column. The column was then thoroughly cleaned using 100% chloroform and a 1:1 chloroform: methanol ratio. 

Using a rotary evaporator, the resultant fractions were concentrated at 40 °C. They were then put into all-glass sample vials previously weighed, sealed with a glass stopper, and labeled. To determine the phytochemicals in each fraction extracted from the solvent systems, as well as to evaluate the purity of each fraction, Thin Layer Chromatography (TLC) was performed.

### Experimental animals

In this investigation, 48 Swiss male mice weighing between 22 and 30 g were employed. The animals were purchased from the University of Ibadan’s Central Animal Facility. They were housed under standard conditions, fed a standard animal diet, and had no restrictions on getting clean drinking water *ad libitum*. The mice were cared for following the 3Rs (replacement, reduction, and refinement) of animal experimentation and environmental enrichments to minimize discomfort and pain. The University of Ibadan Animal Care and Use Research Ethics Committee-approved experimental protocols were adhered to in this work. The study’s animal usage has been approved with ethical approval number UI-ACUREC/072-1222/22.

### Experimental design

*Animal grouping*

1. Control — Animals in this group were given 10 ml/kg of distilled water orally

2. AD + negative control — Scopolamine group receiving only scopolamine (1 mg/kg, IP)

3. A.D. + Crude *A. muricata* extract 25 mg/kg

4. A.D. + Crude *A. muricata* extract 50 mg/kg

5. A.D. + Crude *A. muricata* extract 100 mg/kg

6. A.D. + Donepezil 5 mg/kg

7. AD + fraction with highest AchE activity at 25 mg/kg

8. AD + fraction with highest AchE activity at 100 mg/kg

### Measurement of cognitive performance

All behavioral tests were conducted in the morning to prevent variations in locomotor activity and other factors. The animals are also brought into the test room 30 min before aid animals get familiar with the room. Previous research has shown that while some test variables resist test order, others are sensitive (13).

*Y-maze test (YMT)*

The Y-maze test was used to evaluate the impact of AMME and AMAMF on cognitive impairment as a measure of spatial memory failure. To conduct the test, each animal (mice) was placed in an apparatus consisting of three identical arms (designated A, B, and C) that were symmetrically separated at a 120° angle. When an animal enters each of the three arms consecutively (ABC, CAB, or BCA but not BAB), it is exhibiting alternation behavior. Rats were said to exhibit spontaneous alternation performance when they could visit an arm option other than the one they had visited before while attempting to recall the proper order to visit the arms. (Total alternation number/Total number of entries – 2) X 100 was the formula used to compute the percentage of correct alternation performance, measuring spatial working memory. To eliminate lingering smells from the preceding animal, 70% ethanol was used to sanitize the maze after each test session.

*Measurement of locomotion*

In an open field chamber, the impact of AMME on locomotion was evaluated using counts of horizontal and vertical beam breaks. Sensors that measure vertical beam breaks and count horizontal beam breaks every five minutes were used to detect activity. After every evaluation, the observation cage was cleaned with 70% ethanol to remove scent clues from the preceding animal.

*Novel object recognition test (NORT)*

After five minutes in the empty, open field, the animal had become accustomed to the observation chamber and was allowed to wander. Two identical objects (A and B) with a red pattern were positioned symmetrically 8 cm from the chamber walls and 34 cm apart four hours after the habituation period. The rat was given five minutes to investigate the items in the room after being positioned equally apart from both objects and facing the other way. After training, the rat was put back in its cage. The novel item C, which will feature red and green patterns, was used instead of the red pattern object B during the test session (24 hr later). After being put back in the box, the rat was given five minutes to freely investigate objects A and C. The amount of time the rat spent examining each object was noted. The act of an animal directing its nose directly toward an object at a distance of less than two centimeters and/or making contact with it using its nose and whiskers is known as object exploration. The animal was not allowed to climb or sit on the object during exploration. After every trial, 70% ethanol was used to clean the chamber and the objects to eliminate smell cues. To gauge how well people recognize Object A, the discrimination index (DI) was computed using the formula DI = (Time exploring object A/Time exploring both Objects A and C) x 100.

*Morris water maze*

The Morris water maze is popular for researching learning and spatial memory. The animals were placed in an opaquely colored pool of water (made with nonfat milk powder or nontoxic tempera paint), and they had to swim to a secret platform where they could escape. The animals are in opaque water, so they are unable to see the platform and are unable to use scent to locate the way out. They have to rely on extra-maze or external signals instead. The animals discover the platform faster as they get more accustomed to the activity. 

### Biochemical assays

A modified Jollow method, as reported by Afolabi *et al.* (14), was used to quantify the reduced glutathione (GSH) concentration in aliquots of brain supernatant. The technique of Oladele *et al.*. (15) was used to estimate the tissue levels of MDA, SOD, GST, nitrite, and serum MPO. ELISA kit was used to quantify blood concentration of arginase, IL-6, and TNF-α following standard methods supplied by Elabscience Biotechnology. 

*Determination of acetylcholinesterase activity*

In a colorimetric experiment, the acetylcholinesterase enzyme activity was evaluated in the striatum, prefrontal cortex, and hippocampus using Ellman’s reagent and an enzyme substrate. Briefly, 5,5’-dithio-bis(2-nitrobenzoic acid) (DTNB) was combined with a five-fold supernatant dilution and incubated for 10 min at room temperature. Before incubation with acetylthiocholine iodide (25 µl, 0.028 M) for 3 min and the final absorbance measurement, the mixture’s initial absorbance was measured at 412 nm. Acyl-cholinesterase activity was measured and represented as μmol min^−1^ mg^−1^ tissue based on the change in absorbance per minute (16). 

*Determination of glutamic acid decarboxylase*

The reagents used for this protocol include sodium acetate, Bromocresol green, pyridoxal 5-phosphate, glutamate, and brain supernatant. The stock solution of sodium acetate (20 mM, pH 4.8) was prepared. The assay was founded on the observation that when protons are used during the enzyme-catalyzed reaction, bromocresol green’s color changes due to increased pH. Because bromocresol green and the acetate buffer have similar pK(a), it was used as the indicator. As the reaction progressed, the corresponding absorbance change at 620 nm was measured using a microplate reader. This approach successfully allowed the assessment of reaction kinetic parameters and the detection of improvements in enzymatic activity with a low coefficient of variation despite a discrepancy in the pH of the enzyme preparation and the ideal pH for GAD activity of 2.5.

### Statistical analysis

The statistical program GraphPad® Prism 8.1 was used to examine the data. Tukey’s multiple comparison tests were used for *post hoc* analysis after one-way analysis of variance (ANOVA) was used to compare the test doses with the control group. The typical medication, donepezil, was also compared with test doses. Every outcome was presented as the average ± standard error of the mean (SEM). *P-*values<0.05, or less than 0.05, were considered significant. The data collected over a few days and subjected to Two-way ANOVA analysis were the exception. Appropriate tables and figures were employed to display the data.

## Results

### In vitro screening assays on Annona muricata crude and fractions

*Effect of the crude extract and aqueous methanol fraction on AChE*

The crude extract AMME and aqueous methanol fraction AMAMF consistently showed the highest activity. Both are consistently seen to have the greatest percentage of inhibitory activity and inhibit 50% of the AchE enzymes with the lowest concentration (having the highest activity).

*Ability of the crude extract and aqueous methanol fraction to scavenge radicals in the DPPH assay*

AMME and AMAMF showed the highest DPPH free radical scavenging activity (%), behind the standard Ascorbic acid. The plot of the mean ± SEM shows the AMME has the second-highest activity after ascorbic acid.

### Effect of AMME and AMAMF on short-term working memory in YMAZE

The control group (vehicle) showed the highest number of correct alternations, and the low-dose AMAMF was the only group that showed a significant increase in the number of correct alternations, as seen in Figure 6A. The high-dose crude extract showed the highest incorrect alternation mean ± SEM with significance (**P<0.01) (Figure 6B). From Figure 6C, the control and AMME 100 mg/kg had the highest means ± SEM value. Figure 6D showed a significant difference (^*P*<0.05) between the negative control and vehicle group.

### Effect of AMME and AMAMF on spatial memory using the Morris water maze

For the duration of the target quadrant visit, the Donepezil group and the highest dose of the fraction were significant when compared with the means of the other groups. The Donepezil group showed the highest activity with a *P*-value less than 0.001. The comparison of the negative control group versus the vehicle group showed a great significance at a *P*-value of 0.001 (Figure 7A). The mean value of the treatment groups for Donepezil, AMME 25 mg/kg, and 100 mg/kg showed statistical significance (Figure 7B). The latency to the platform in Figure 7C showed a reduced time till latency in the donepezil and 100 mg/kg dose of AMAMF. Similarly, Figure 7E demonstrated a lower delay to the target quadrant for the Donepezil group. The time taken to escape latency was plotted and analyzed with two-way ANOVA, with the medium dose of the fraction being the highest and other treatment groups being less. 

### Effect of AMME and AMAMF on locomotor activity and discrimination index in open field test and novel object recognition test

In Figure 8A, the plot of the NORT discrimination index showed that the fraction’s highest dose improves memory, although not statistically significant. The locomotor activity is measured by counting the line crossings, as seen in Figure 8B. The AMME 25 mg showed a high level of line crossing.

### Anti-oxidant and oxidative stress assays

The concentration of MDA in the mouse hippocampal region was significantly (*P*<0.05) raised by scopolamine (1 mg/kg, IP) (Figure 9A & B). The level of MDA and nitrite was significantly suppressed in the hippocampal region of treatment mice. The treated mice also revealed a slight but significant reduction in the MDA and nitrite level of the prefrontal cortex when contrasted with the negative control mice (Figure 9A & B). Scopolamine administration reduced the concentration of GSH and GST in the hippocampus significantly (*P*<0.05) when compared with the vehicle (Figure 9C & D). The three doses of AMME enhanced the hippocampal GSH and GST levels significantly. The crude extract and fraction inexplicably increased the prefrontal cortex tissue levels of GSH and GST significantly, as seen in Figure 9H& I. The hippocampal nitrite level was raised in the memory-impaired mice, unlike in vehicle control mice, and the low doses of crude extract and fraction reduced the tissue level significantly (Figure 9B). All treatment doses of crude extract and fractions significantly reduced the tissue nitrite levels in the prefrontal cortex (Figure 9G). The reduced SOD level in the prefrontal and hippocampus tissue of the negative control mice was significantly reversed in the treatment of mice with varying doses of AMME, AMAMF, and Donepezil (Figures 9E & J). 

### Neurotransmitter-related enzymes biochemical assays

Acetylcholinesterase (AchE) activity in the prefrontal cortex and hippocampus

All groups show a reduction in AChE activity in the hippocampus, with the group receiving AMME 25 mg/kg dose being the only one showing any significance (*P*<0.05) (Figure 10A). In the prefrontal cortex (Figure 10B), all groups show a reduction in AChE activity, with the group receiving donepezil at 5 mg/kg and AMME 50 mg/kg dose being the only one showing any significance (*P*<0.05 and *P*<0.01, respectively). Compared with the vehicle group, the negative control group also showed statistical significance (Figures 10A & B).

Glutamic acid decarboxylase (GAD) activity in the prefrontal cortex and hippocampus

All groups show an increase in GAD activity in the hippocampus, with the Donepezil, AMME 25 mg/kg, AMME 100 mg/kg, AMAMF 25, and 100 mg/kg showing significance (*P*<0.05) increase (Figure 10C). In the prefrontal cortex, there was a statistical increase in the activity of GAD in the Donepezil group and groups receiving the higher doses of the crude extract and the fractions (Figure 10D).

### Inflammatory-related enzymes and mediators biochemical assays

The treatment and scopolamine-induced memory impairment had no significant effect on the hippocampal and prefrontal cortex tissue level of MPO (Figures 11 A & B). Elevated hippocampal level of arginase in the negative control mice was significantly reduced in the treatment group, especially AMME 50 mg/kg (*P*<0.01), AMMF 25 mg/kg (*P*<0.05), and Donepezil (*P*<0.05) (Figure 11C). The arginase level was significantly reduced across the prefrontal cortex (Figure 11D). Figure 11 E shows a slight reduction in the hippocampal TNF-alpha level in all treatment groups. However, a significant reduction was only observed in the AMME 50 mg/kg and AMAMF 100 mg/kg group. Both the crude extract and fractions show a significant reduction in TNF-α levels in the prefrontal cortex of mice (Figure 11F). Also, treatment mice showed a significant decrease in IL-6 levels in the tissue of the hippocampus (Donepezil; *P*<0.05, AMME 25 mg/kg; *P*<0.05, AMME100 mg/kg *P*<0.01, and both doses of AMAMF; *P*<0.05). Both the highest dose of the crude extract and fractions show a significant reduction (*P*<0.05) in the IL-6 levels of the prefrontal cortex (Figure 11G & H).

### Histological evaluation

Figure 12 A-H shows the photomicrograph of a brain hippocampus and frontal lobe stained by cresyl fast violet. There was a normal distribution of nissl bodies within the hippocampal neuronal cells of the vehicle group (Figure 12A). The prefrontal cortex of the vehicle group displays normal neuronal cells with normal nissl body cytoplasmic distribution. (Figure 12I). In the negative control group, the hippocampus shows a poor distribution of nissl bodies within the neuronal cells, and the prefrontal cortex shows an abnormal cytoplasmic distribution of nissl bodies (Figures 12B & J). Donepezil group; there was a normal nissl body cytoplasmic distribution within the neuronal cells of the hippocampus with few mildly depleted neurons, and the prefrontal cortex shows many normal neural cells with a typical distribution of nissl bodies in the cytoplasm. However, few neurons show poor nissl distribution within their cytoplasm (Figures 12C & K). The hippocampus and prefrontal cortex of 25 mg/kg AMME show abnormal cytoplasmic distribution of nissl bodies in a few neurons while the majority of neurons are normal (Figures 12D & L). The hippocampus and prefrontal cortex of 50 and 100 mg/kg AMME, 25 100 mg/kg AMAMF treated mice show a well-organized, normal neuronal cell with a normal cytoplasmic distribution of nissl bodies. The 100 mg/kg AMAMF-treated mice show a normal distribution of nissl bodies within the neuronal cells but are structurally poorly organized (Figure 12H).

## Discussion

An imbalance between anti-oxidant and reactive oxygen species (ROS) results in oxidative stress, the hallmark of several ailments, including neurodegenerative diseases. Methanol extract of *A*. *muricata *exhibits potent anti-oxidant activities with high DPPH scavenging potential; this shows that methanol extracts have a powerful ability to prevent the formation of or remove free radicals. Therefore, it can provide redox functions protons to unstable free radicals; this action is instrumental in normalizing the damaging effects of free radicals in the body system to alleviate diseases (17). 

Scopolamine significantly decreased the level of glutathione S. transferase (GST), glutathione (GSH), and superoxide dismutase (SOD). However, it significantly raised nitrite and malondialdehyde (MDA) levels in both hippocampal and prefrontal cortex tissue homogenate of experimental mice. Because of the generation of reactive oxygen species and the depletion of various anti-oxidant enzymes, there is a correlation between the rise in oxidative stress and the cognitive deterioration linked to AD (18, 19). However, A. *muricata* extract causes an increase in SOD, GST, and GSH levels to mitigate the impact of scopolamine.

Postsynaptic neuromuscular connections include the cholinergic enzyme and neuromodulator AChE, mostly in nerves and muscles. Acetylcholine (ACh), a neurotransmitter, is easily catalyzed into acetic acid and choline (20). Acetylcholinesterases (AChE) stop synapses from communicating with one another and from spreading ACh and activating surrounding receptors. The AChE is the primary target when combating cholinergic dysfunction associated with AD; this is done by employing a cholinesterase inhibitor. Reports have shown that scopolamine, a muscarinic receptor agonist, causes an increase in extracellular ACh in the brain (20). In one set of mice in this study, scopolamine was used to generate an elevation in AChE concentration in both the prefrontal cortex and the hippocampus. In this case, neuronal transmission and synaptic transmission between the brain neurons will be impaired, which could result in loss of cognitive function. However, cognitive impairment caused by scopolamine was improved by *A. muricata* extracts at 50 and 100 mg/kg of AMME, suggesting that the extracts at the reported dosage can improve cognitive function.

The enzyme glutamic acid decarboxylase (GAD) transforms the excitatory neurotransmitter glutamate into Ƴ-aminobutyric acid, which is an inhibitory neurotransmitter that regulates neurotransmission in the central nervous system (CNS) (21). In this study, the GAD level was significantly reduced by scopolamine. GAD is a muscarinic receptor that enhances neuronal excitability and synaptic transmission, and scopolamine has an antagonistic effect on muscarinic receptors, which could alter synaptic and neuronal activities in the brain, leading to cognitive impairment (22). This study found that the inhibiting effects of scopolamine were ameliorated in the treated groups in both organs. This suggests that *A. muricata* helps to improve brain function and cognition.

It has been established by studies that scopolamine results in the alteration of synaptic functions between neurons, leading to neurological disorders such as AD, majorly in the neurons of the hippocampus as well as working memory (23, 24). The percentage of spontaneous alteration was decreased in Y-maze activity (15), and this result was supported by the results obtained from short-term working memory of the Y-MAZE and the spatial working memory using the Morris water maze in the current study. Our result shows that impairment in the cognitive ability of short-term memory as a result of scopolamine was mitigated by *A. muricata*, which shows that it is instrumental in the enhancement of cognitive function.

Several studies have revealed that inflammatory cytokines like interleukin-6 (IL-6) and tumor necrosis factor-alpha (TNF-alpha) significantly affect memory impairment. TNF-alpha and IL-6, known for their roles in immune response and inflammation, also influence cognitive functions, particularly memory. Their dual roles highlight a complex interplay between promoting and impairing cognitive abilities, illustrating the multifaceted impact of cytokines on brain function.

Interleukin 6 (IL-6) and tumor necrosis factor-alpha (TNF-alpha) are important pro-inflammatory cytokines implicated in various immune responses and the regulation of inflammation. While their primary roles are within the immune system, emerging research has highlighted their significant impact on cognitive functions, particularly memory processes. TNF-alpha and IL-6 are pivotal in mediating the body’s response to injury and infection and play complex roles in neural plasticity, brain function, and the pathophysiology of neurodegenerative diseases. These cytokines’ involvement extends beyond traditional immunological boundaries, implicating them in the intricate interplay between inflammation and cognitive health, where they can influence both beneficial and detrimental outcomes on memory formation and recall.

Utilizing a transgenic mouse model that overexpresses glial fibrillary acidic protein-interleukin 6 (GFAP-IL6) to simulate chronic neuroinflammation, the study by Chesworth *et al.* (25) delves into the complex relationship between neuroinflammation, microglial activation, and cognitive function. It investigates age-related cognitive decline and assesses the potential therapeutic role of apigenin, a natural flavonoid, in mitigating these effects. Despite observing a reduction in microglial activation with chronic apigenin treatment, the research reports no significant improvement in spatial memory performance in GFAP-IL6 mice. This highlights the nuanced interaction between neuroinflammation and cognitive function and suggests that apigenin’s ability to reverse cognitive impairments may be limited within the context of chronic neuroinflammation. The findings emphasize the need for further exploration of neuroinflammatory pathways and the role of microglia in cognitive functions to develop more effective therapeutic strategies for neurodegenerative diseases associated with chronic inflammation.

In our study, we explored the effects of various treatments on the levels of inflammatory-related enzymes and mediators such as myeloperoxidase (MPO), arginase, TNF-alpha, and IL-6 in mice’s hippocampal and prefrontal cortex tissues. The findings revealed no significant changes in MPO activity, paralleling the results of Quilez *et al.* (26), who documented a marked inhibition of MPO activity in inflamed tissues following treatment with *A. muricata* leaf extract. This comparison underscores the potential anti-inflammatory properties of the treatments used in our study, similar to those observed with *A. muricata*, suggesting a consistent pattern of inflammatory response modulation across different experimental setups.

Further, our results demonstrated a reduction in TNF-alpha and IL-6 levels, particularly significant in groups treated with higher doses. This outcome correlates well with the findings from Al Omairi *et al.* (27), where soursop fruit extract was shown to mitigate scopolamine-induced amnesia and oxidative stress, implicating the cholinergic and Nrf2/HO-1 pathways in the anti-inflammatory response. The decrease in pro-inflammatory cytokines in our study suggests a similar biochemical pathway could be active, highlighting a potentially universal mechanism by which these treatments exert neuroprotective effects through inflammation modulation.

Moreover, the significant reduction in IL-6 levels observed in our study aligns with the discussions in Bourgognon and Cavanagh (28), where IL-6 is portrayed as a dual-role player in brain function, capable of both supporting and impairing cognitive processes depending on its levels. This correlation is particularly compelling, offering a broader understanding of how cytokine modulation might interact with cognitive functions and suggesting that the alterations in cytokine levels we observed could directly impact memory and learning processes in treated mice (28). Collectively, these correlations with existing research validate our findings and contribute to a deeper understanding of the inflammatory pathways involved in neuroprotection and cognitive function. This discussion supports the notion that targeting inflammatory mediators like IL-6 and TNF-alpha can be a viable strategy for managing neurodegenerative conditions associated with cognitive decline. 

Likewise, in our histological evaluations, we observed that treatments such as Donepezil and AMME significantly restored normal neuronal structure in the hippocampus, particularly the distribution of Nissl bodies, which were poorly distributed in the negative control group. This poor distribution is indicative of neuronal stress or damage, as similarly noted in the study by Yoon *et al.* (29), where scopolamine-induced cognitive impairment led to decreased Nissl substance, underscoring the importance of treatments that can counteract such effects to maintain neuronal integrity (29). Furthermore, Etibor *et al.* (30) reported that exposure to Datura metel, known for its neurotoxic effects, reduced Nissl substance and overall neuronal degradation, which aligns with our findings in the negative control group, suggesting a similar pathological condition. Our findings that AMME treatment can improve the distribution and integrity of Nissl bodies suggest a protective or reparative effect against neuronal stress or damage, highlighting their potential therapeutic implications in neurodegenerative conditions.

**Figure 1:**
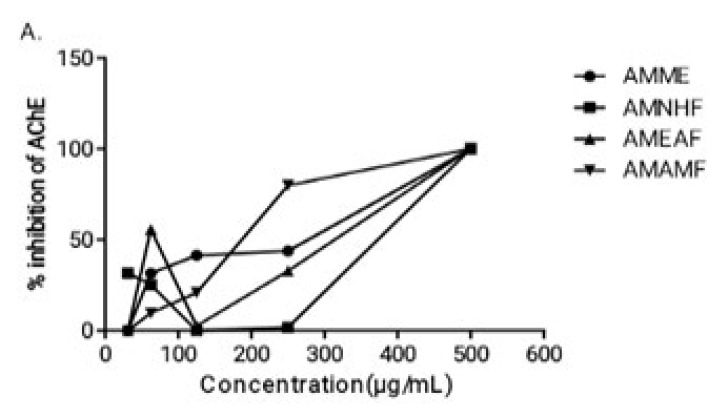
Percentage inhibition of acetylcholinesterase enzyme* in vitro*

**Figure 2:**
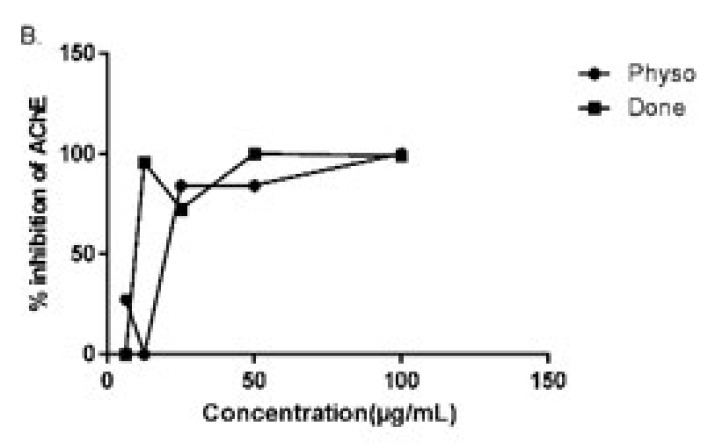
Percentage inhibition of donepezil and physostigmine on acetylcholinesterase enzyme *in vitro*

**Figure 3:**
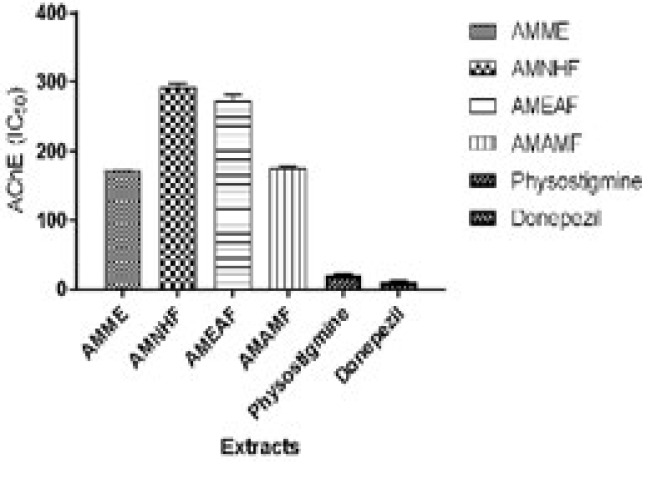
Effects of the *Annona muricata* crude extract, fractions, Physostigmine, and Donepezil on AChE (IC_50_) *in vitro*

Every bar depicts the Mean ± SEM for six animals in each category. Comparing these values to the group that received physostigmine and donepezil reveals significance at *P*<0.05 (ANOVA followed by Tukey *post hoc* test) when treated with AMME, AMNHF, AMEAF, and AMAMF

AMME: *Annona muricata* methanol extract; AMNHF: *Annona muricata* n-hexane fraction; AMEAF: *Annona muricata* ethyl-acetate fraction; AMAMF: *Annona muricata* aqueous methanol fraction

**Figure 4:**
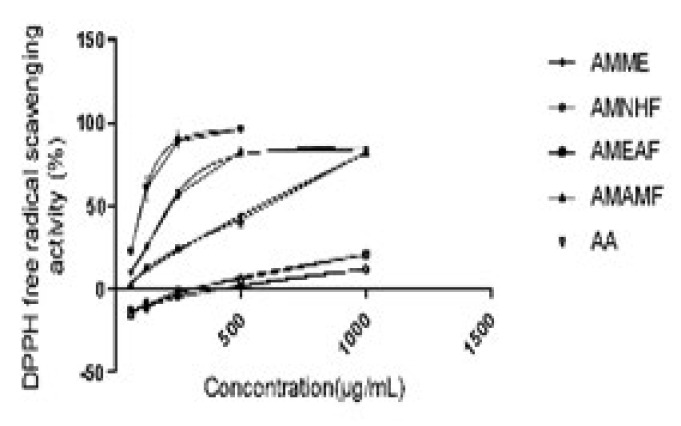
Radical scavenging activity of AMME, AMNHF, AMAMF, and ascorbic acid

AMME: *Annona muricata* methanol extract; AMNHF: *Annona muricata* n-hexane fraction; AMEAF: *Annona muricata* ethyl-acetate fraction; AMAMF: *Annona muricata* aqueous methanol fraction

**Figure 5:**
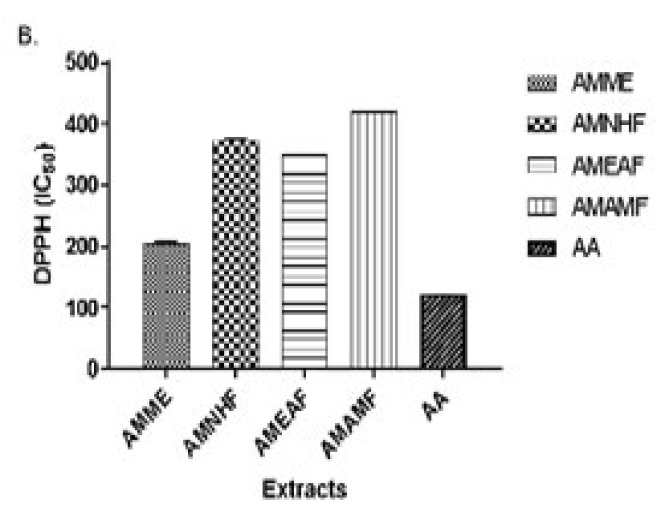
IC_50_ of AMME, AMNHF, AMAMF, and ascorbic acid

AMME: *Annona muricata* methanol extract; AMNHF: *Annona muricata* n-hexane fraction; AMEAF: *Annona muricata* ethyl-acetate fraction; AMAMF: *Annona muricata* aqueous methanol fraction

**Figure 6A-D:**
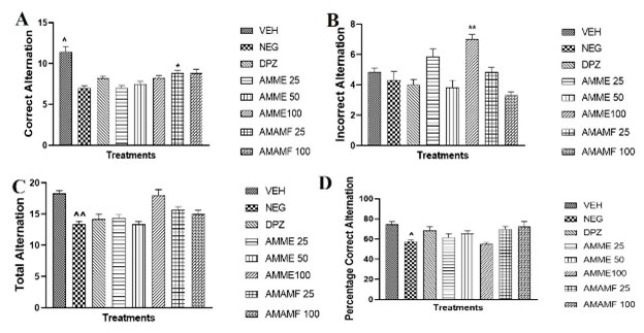
The neurobehavioural effect of *Annona muricata* methanol extract and aqueous methanol fraction

Every bar depicts the Mean ± SEM for six animals in each category. When compared to the scopolamine (SCOP) group, these values are significant at *P<0.05 (ANOVA followed by Tukey *post hoc* test). And ^*P*<0.05 when contrasted with the control group (VEH) using an ANOVA and Tukey's *post hoc* test, the treated groups received 25, 50, and 100 mg/kg of AMME crude extract, 25 mg/kg of AMAMF, and 5 mg/kg of the cholinesterase inhibitor donepezil (DPZ). (*P<0.05, **P<0.01, ***P<0.001, compared to the SCOP Model group; ^*P*<0.05, ^^*P*<0.01, ^^^*P*<0.001, compared to the Control group).

**Figure 7. A-E:**
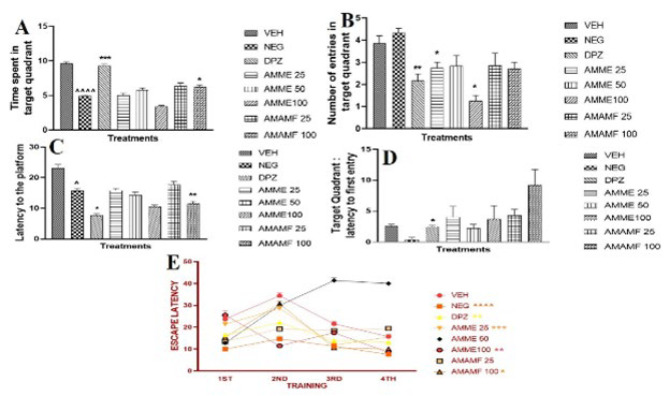
The neurobehavioural effect of Annona muricata methanol extract and aqueous methanol fraction

Every bar depicts the Mean ± SEM for six animals in each category. These values, as compared to the scopolamine (SCOP) group (ANOVA followed by Tukey *post hoc* test), are significant at *P<0.05. ^*P*<0.05 in comparison to the VEH control group (ANOVA followed by Tukey's *post hoc* test) and the treated 25, 50, and 100 mg/kg crude extract of AMME and 25 mg/kg of AMAMF, Donepezil (DPZ) 5 mg/kg - a cholinesterase inhibitor. (*P<0.05, **P<0.01, ***P<0.001, vs SCOP Model group; ^*P*<0.05, ^^*P*<0.01, ^^^*P*<0.001, vs Control).

**Figure 8. A-B:**
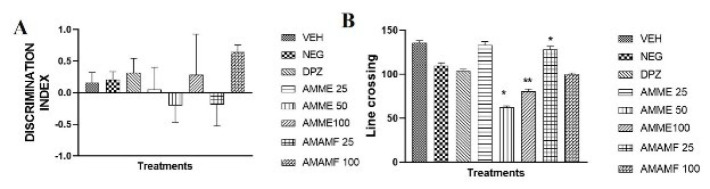
The neurobehavioural effect of Annona muricata methanol extract and aqueous methanol fraction

Every bar depicts the Mean ± SEM for six animals in each group. These values, as compared to the scopolamine (SCOP) group (ANOVA followed by Tukey *post hoc* test), are significant at *P<0.05. ^*P*<0.05 when compared with the control group (VEH) (ANOVA followed by Tukey's *post hoc *test) and the treated 25, 50, and 100 mg/kg crude extract of AMME and 25 mg/kg of AMAMF, Donepezil (DPZ) 5 mg/kg - a cholinesterase inhibitor. (*P<0.05, **P<0.01, ***P<0.001, vs SCOP Model group; ^*P*<0.05, ^^*P*<0.01, ^^^*P*<0.001, vs Control).

**Figure 9. A-E:**
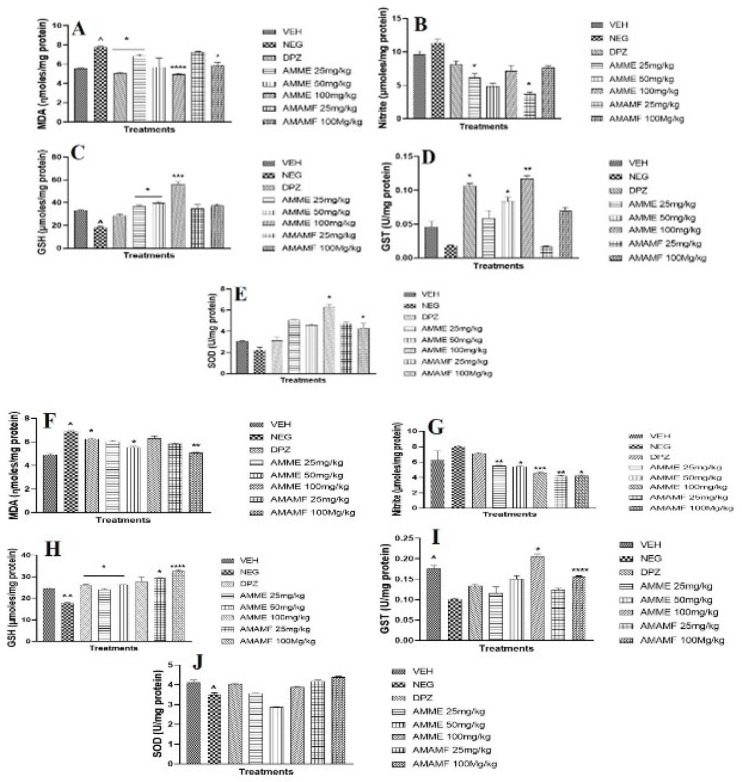
The effect of AMME and AMAMF on hippocampal tissue MDA, nitrite, GSH, GST, SOD (A-E) and prefrontal cortex MDA, nitrite, GSH, GST, SOD **(F-J)**

Each bar represents the Mean ± SEM for 6 animals per group.These values are significant at *P<0.05 when compared with the scopolamine (SCOP) group (ANOVA followed by Tukey *post hoc* test). ^*P*<0.05 when compared with the control group (VEH) (ANOVA followed by Tukey's *post hoc *test) and the treated 25, 50, and 100 mg/kg crude extract of AMME and 25 mg/kg of AMAMF, Donepezil (DPZ) 5 mg/kg - a cholinesterase inhibitor. (*P<0.05, **P<0.01, ***P<0.001, vs SCOP Model group; ^*P*<0.05, ^^*P*<0.01, ^^^*P*<0.001, vs Control)

**Figure 10.A-D:**
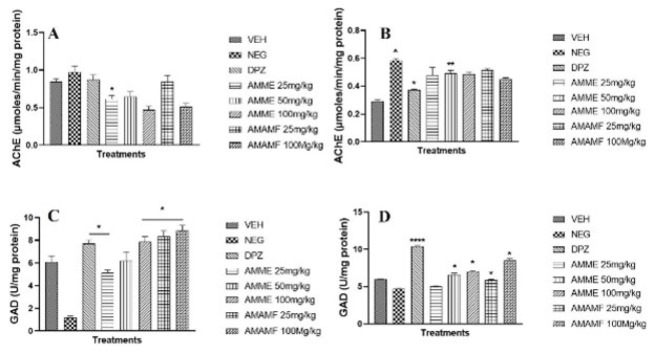
The effect of AMME and AMAMF on hippocampal and prefrontal tissue acetycholinesterase and glutamic acid decarboxylase

Each bar depicts the Mean ± SEM for six animals in each group. When compared to the scopolamine (SCOP) group, these values are significant at *P<0.05 (ANOVA followed by Tukey *post ho*c test). ^*P*<0.05 in comparison to the VEH control group (ANOVA with Tukey's *post hoc* test) and the treated 25, 50, and 100 mg/kg crude extract of AMME and 25 mg/kg of AMAMF, Donepezil (DPZ) 5 mg/kg - a cholinesterase inhibitor. (*P<0.05, **P<0.01, ***P<0.001, vs SCOP Model group; ^*P*<0.05, ^^*P*<0.01, ^^^*P*<0.001, vs Control).

**Figure 11. A-H:**
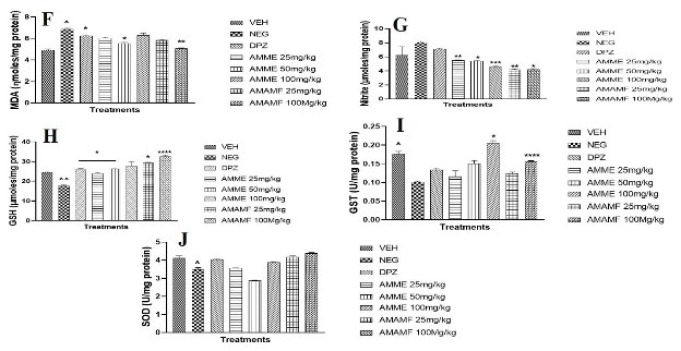
The effect of AMME and AMAMF on hippocampal MPO (A), Arginase (C), TNF-α (E), IL-6 (G) and prefrontal tissue MPO (B) Arginase (D), TNF-α (F), IL-6 (H)

Every bar depicts the Mean ± SEM for six animals in each category. When compared to the scopolamine (SCOP) group, these values are significant at *P<0.05 (ANOVA followed by Tukey* post hoc* test). ^*P*<0.05 when compared with the control group (VEH) (ANOVA followed by Tukey's *post hoc *test) and the treated 25, 50, and 100 mg/kg crude extract of AMME and 25 mg/kg of AMAMF, Donepezil (DPZ) 5 mg/kg - a cholinesterase inhibitor. (*P<0.05, **P<0.01, ***P<0.001, vs SCOP Model group; ^*P*<0.05, ^^*P*<0.01, ^^^*P*<0.001, vs Control).

**Figure 12:**
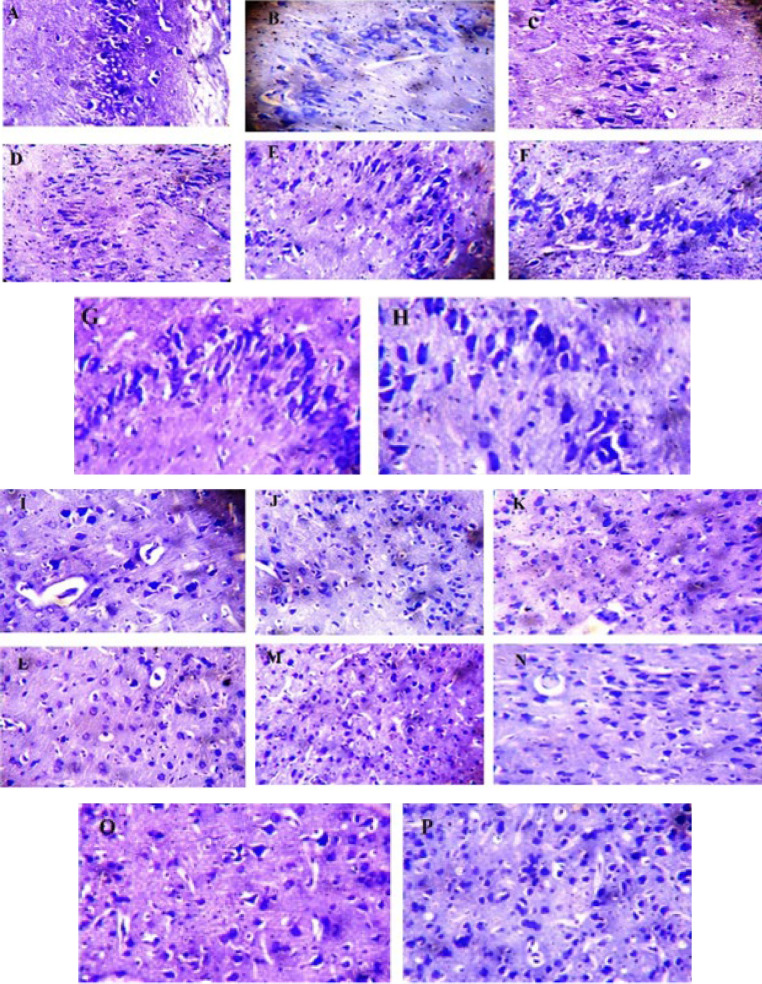
Photomicrograph of the hippocampal (A-H) and prefrontal tissue (I-P) of male mice stained by cresyl fast violet

A represents vehicle control; B represents negative control; C represents the donepezil group; D-F represents 25, 50, and 100 mg/kg AMME treatment group; G-H represents 25 and 100 mg/kg AMAMF treatment groups; I represent vehicle control; J represents negative control; K represents the donepezil group; L-N represents 25, 50, and 100 mg/kg AMME treatment group; O-P represents 25 and 100 mg/kg AMAMF treatment groups.

## Conclusion

The findings of the research indicate that *A. muricata* appears to enhance memory recovery, as evidenced by behavioral models and the histological analysis of the hippocampus, along with the observed neuronal alterations in the prefrontal cortices of the mice. The study showed the neuroprotective effects of AMME and AMAMF in memory impairment models. The extracts showed potent AChE inhibition and positive effects on memory and anti-oxidant enzyme levels.

## Declaration

All authors declare that no AI tool was used for the preparation of this manuscript.

## References

[1] Bhushan I, Kour M, Kour G, Gupta S, Sharma S, Yadav A (2018). Alzheimer’s disease: Causes & treatment–A review. Ann Biotechnol.

[2] Oyeleke MB, Owoyele BV (2022). Saponins and flavonoids from Bacopa floribunda plant extract exhibit antioxidant and anti- inflammatory effects on amyloid beta 1-42-induced Alzheimer’s disease in BALB/c mice. J Ethnopharmacol.

[3] Nazir N, Zahoor M, Nisar M, Karim N, Latif A, Ahmad S (2020). Evaluation of neuroprotective and anti-amnesic effects of Elaeagnus umbellata thunb on scopolamine-induced memory impairment in mice. BMC Complement Med Ther.

[4] Goldstein G (2011). Delirium, dementia and amnestic and other cognitive disorders (neurocognitive disorders). Adult Psychopathol Diagn.

[5] Tönnies E, Trushina E (2017). Oxidative stress, synaptic dysfunction, and Alzheimer’s disease. J Alzheimers Dis.

[6] Meneses A (2014). Neurotransmitters and memory: Cholinergic, glutamatergic, gabaergic, dopaminergic, serotonergic signaling and memory. Identification of Neural Markers Accompanying Memory.

[7] Daulatzai MA (2010). Early stages of pathogenesis in memory impairment during normal senescence and Alzheimer’s disease. J Alzheimers Dis.

[8] Cheng YJ, Lin CH, Lane HY (2021). Involvement of cholinergic adrenergic and glutamatergic network modulation with cognitive dysfunction in Alzheimer’s disease. Int J Mol Sci.

[9] Gavamukulya Y, Wamunyokoli F, El-Shemy HA (2017). Annona muricata: Is the natural therapy to most disease conditions including cancer growing in our backyard? A systematic review of its research history and future prospects. Asian Pac J Trop Med.

[10] Mutakin M, Fauziati R, Fadhilah FN, Zuhrotun A, Amalia R, Hadisaputri YE (2022). Pharmacological activities of soursop (Annona muricata Lin). Molecules.

[11] Coria-Téllez AV, Montalvo-Gónzalez E, Yahia EM, Obledo- Vázquez EN (2018). Annona muricata: A comprehensive review on its traditional medicinal uses, phytochemicals, pharmacological activities, mechanisms of action and toxicity. Arab J Chem.

[12] Singh D, Hembrom S, Raj A (2019). Neuroprotective effect of flavonoids: A systematic review. J Pharmacogn Phytochem.

[13] Zemdegs J, Quesseveur G, Jarriault D, Pénicaud L, Fioramonti X, Guiard BP (2016). High-fat diet-induced metabolic disorders impair 5-HT function and anxiety-like behavior in mice. Br J Pharmacol.

[14] Afolabi O, Alabi B, Omobowale T, Oluranti O, Iwalewa O (2022). Cysteamine mitigates torsion/detorsion-induced reperfusion injury via inhibition of apoptosis, oxidative stress and inflammatory responses in experimental rat model. Andrologia.

[15] Oladele JO, Oyeleke OM, Olowookere BD, Babatope OD, Olaniyan MD, Akindolie BO, Oladele OT (2021). Bitter leaf (Vernonia amygdalina) modulates nitrobenzene-induced renal damage in rats via suppression of oxido-inflammatory activities. EABR.

[16] Ajayi AM, Ben-Azu B, Onasanwo SA, Adeoluwa O, Eduviere A, Ademowo OG (2019). Flavonoid-rich fraction of ocimum gratissimum attenuates lipopolysaccharide-induced sickness behavior, inflammatory and oxidative stress in mice. Drug Res (Stuttg).

[17] Postua PA, Sadiki FZ, Idrissi ME, Cioanca O, Trifan A, Hritcu L (2019). Pinus halepensis essential oil attenuates the toxic alzheimer’s amyloid beta (1-42)-induced memory impairment and oxidative stress in the rat hippocampus. Biomed Pharmacother.

[18] Gella A, Durany N (2009). Oxidative stress in alzheimer’s disease. Cell Adh Migr.

[19] Zhao Y, Zhao B (2013). Oxidative stress and the pathogenesis of alzheimer’s disease. Oxid Med Cell Longev.

[20] McHardy SF, Wang HYL, McCowen SV, Valdez MC (2017). Recent advances in acetylcholinesterase inhibitors and reactivators: An update on the patent literature (2012-2015). Expert Opin Ther Pat.

[21] Szpręgiel I, Wrońska D, Kmiecik M, Pałka S, Kania BF (2021). Glutamic acid decarboxylase concentration changes in response to stress and altered availability of glutamic acid in rabbit (Oryctolagus cuniculus) brain limbic structures. Animals.

[22] Newman LA, Gold PE (2016). Attenuation in rats of impairments of memory by scopolamine, a muscarinic receptor antagonist, by mecamylamine, a nicotinic receptor antagonist. Psychopharmacology (Berl).

[23] Lu C, Shi Z, Dong L, Lv J, Xu P, Li Y (2017). Exploring the effect of ginsenoside Rh1 in a sleep deprivation-induced mouse memory impairment model. Phytother Res.

[24] Cai P, Fang SQ, Yang XL, Wu JJ, Liu QH, Hong H (2017). Rational design and multibiological profiling of novel donepezil– trolox hybrids against alzheimer’s disease with cholinergic anti-oxidant neuroprotective and cognition-enhancing properties. ACS Chem Neurosci.

[25] Chesworth R, Gamage R, Ullah F, Sonego S, Millington C, Fernandez A (2021). Spatial memory and microglia activation in a mouse model of chronic neuroinflammation and the anti- inflammatory effects of apigenin. Front Neurosci.

[26] Quilez AM, Montserrat-de la Paz S, De la Puerta R, Fernández-Arche MA, García-Giménez MD (2015). Validation of ethnomedicinal use as anti-inflammatory of a decoction from Annona muricata leaves. Afr J Tradit Complement Altern Med.

[27] Al Omairi NE, Al-Brakati AY, Kassab RB, Lokman MS, Elmahallawy EK, Amin HK (2019). Soursop fruit extract mitigates scopolamine-induced amnesia and oxidative stress via activating cholinergic and Nrf2/HO-1 pathways. Metab Brain Dis.

[28] Bourgognon JM, Cavanagh J (2020). The role of cytokines in modulating learning and memory and brain plasticity. BNA.

[29] Yoon WB, Cho HJ, Kim JE, Park JW, Kang MJ, Bae SJ (2018). Comparison of scopolamine-induced cognitive impairment responses in three different ICR stocks. Lab Anim Res.

[30] Etibor TA, Ajibola MI, Buhari MO, Safiriyu AA, Akinola OB, Caxton-Martins EA (2015). Datura metel administration distorts medial prefrontal cortex histology of Wistar rats. World J Neurosci.

